# Supplementary material for: Sepsis awareness and knowledge amongst nurses, physicians and paramedics of a tertiary care center in Switzerland: A survey-based cross-sectional study
Source: PLoS One. 2023 Jun 28;18(6):e0285151. doi: 10.1371/journal.pone.0285151 (PMC10306229; doi:10.1371/journal.pone.0285151)
Supplement: S1 Table — Only variables having a significant effect (p-value ≤ 0.05) are included. (DOCX) [file pone.0285151.s003.docx]

Supplementary table 1. Results of the univariable logistics regression analysis. Only variables having a significant effect (p-value ≤ 0.05) are included.

| Profession | Classes 1/2 | Variable (value) | Outcome | OR | CI 95% | | p-value |
| --- | --- | --- | --- | --- | --- | --- | --- |
|  |  |  |  |  | lower | upper |  |
| Nurses | 256/347 | Ever had training on sepsis | SOFA score knowledge | 5.71 | 2.67 | 12.24 | 0.00 |
|  |  |  | qSOFA score knowledge | 3.54 | 1.93 | 6.50 | 0.00 |
|  | 497/106 | Sepsis formation during the last three years | SOFA score knowledge | 5.30 | 3.04 | 9.22 | 0.00 |
|  |  |  | qSOFA score knowledge | 5.55 | 3.29 | 9.35 | 0.00 |
|  | 465/154 | Sepsis knowledge estimation very good or good | SOFA score knowledge | 4.42 | 2.58 | 7.58 | 0.00 |
|  |  |  | qSOFA score knowledge | 2.90 | 1.75 | 4.79 | 0.00 |
|  | 392/227 | Sepsis management estimation very good or good | SOFA score knowledge | 4.22 | 2.41 | 7.40 | 0.00 |
|  |  |  | qSOFA score knowledge | 2.33 | 1.42 | 3.81 | 0.00 |
| Paramedics | 35/113 | Ever had trianing on sepsis | Paramedic’s knowledge on sepsis management | 5.72 | 1.89 | 17.30 | 0.00 |
|  | 112/36 | Sepsis continuing education during the last three years | qSOFA score knowledge | 3.27 | 1.16 | 9.25 | 0.03 |
|  |  |  | qSOFA items knowledge | 10.09 | 1.02 | 100.3 | 0.05 |
|  |  |  | Sepsis risk factors knowledge | 2.85 | 1.16 | 6.95 | 0.02 |
|  | 131/18 | Sepsis knowledge estimation very good or good | qSOFA score knowledge | 7.70 | 2.45 | 24.23 | 0.00 |
|  |  |  | qSOFA items knowledge | 26.00 | 2.54 | 266.0 | 0.01 |
|  | 120/29 | Sepsis management estimation very good or good | qSOFA score knowledge | 3.50 | 1.20 | 10.19 | 0.02 |
|  |  |  | qSOFA items knowledge | 13.73 | 1.37 | 137.3 | 0.03 |
| physicians | 45/303 | Ever had training on sepsis | qSOFA score knowledge | 2.00 | 1.04 | 3.84 | 0.04 |
|  | 197/151 | Sepsis continuing education the last three years | Sepsis definition knowledge | 2.24 | 1.39 | 3.59 | 0.00 |
|  |  |  | SOFA score knowledge | 4.26 | 2.71 | 6.70 | 0.00 |
|  |  |  | qSOFA score knowledge | 3.36 | 2.15 | 5.24 | 0.00 |
|  |  |  | qSOFA items knowledge | 2.58 | 1.45 | 4.60 | 0.00 |
|  | 139/209 | Medical experience of more than five years | SOFA score knowledge | 0.41 | 0.26 | 0.63 | 0.00 |
|  |  |  | qSOFA score knowledge | 0.37 | 0.24 | 0.58 | 0.00 |
|  | 227/121 | Sepsis knowledge estimation very good or good | SOFA score knowledge | 2.43 | 1.55 | 3.83 | 0.00 |
|  |  |  | qSOFA score knowledge | 1.86 | 1.19 | 2.92 | 0.01 |
|  |  |  | qSOFA items knowledge | 4.10 | 2.29 | 7.37 | 0.00 |
|  | 205/143 | Sepsis management estimation very good or good | SOFA score knowledge | 2.45 | 1.58 | 3.80 | 0.00 |
|  |  |  | qSOFA score knowledge | 2.07 | 1.34 | 3.19 | 0.00 |
|  |  |  | qSOFA items knowledge | 3.80 | 2.09 | 6.91 | 0.00 |
