# Supplementary material for: Sepsis awareness and knowledge amongst nurses, physicians and paramedics of a tertiary care center in Switzerland: A survey-based cross-sectional study
Source: PLoS One. 2023 Jun 28;18(6):e0285151. doi: 10.1371/journal.pone.0285151 (PMC10306229; doi:10.1371/journal.pone.0285151)
Supplement: S1 File — (DOCX) [file pone.0285151.s004.docx]

**Survey translation, nurses version

- Demographics**

1. What year were you born in?
Single choice with years from 1950 to 2004.

2. What is your gender?
Single choice: Male / Female/ Other.

3. How many years have you worked in healthcare?
Single choice: < 1 year / from 1 to 3 years / from 3 to 5 years / from 5 to 10 years / from 10 to 15 years / more than 15 years.

4. What is your profession?
Single choice: Nurse / Physician / Paramedic.

5. What is your grade?
Single choice: Graduate / Head of unit / Head of service / Clinician nurse / Specialized clinician nurse / Trainer / Other.

6. In what service do you work?
Single choice: Emergency Department / Intensive Care Unit / Internal Medicine / Endocrinology and Diabetology / Gastroenterology and Hepatology / Geriatrics / Immunology and Allergology / Genetic Medicine / Infectious Diseases / Nephrology / Pneumology / Cardiology / Angiology / Hematology / Rheumatology / Neurology / Neuropsychology and Neurorehabilitation / Memory Center / Palliative Care / Organ Transplant Center / Sleep Center / Dermatology and Venerology / Preventive Hospital Medicine / Anesthesiology / Cardiac Surgery / Thoracic Surgery / Visceral Surgery / Vascular Surgery / Septic Surgery / Orthopedics and Traumatology / Plastic and Hand Surgery / Rehabilitation / Neurosurgery / Urology / Ear-Nose-Throat / Surgical Research / Obstetrics and Gynecology / Oncology / Radio-oncology / Immuno-oncology / Radio diagnostic and Interventional Radiology / Psychiatry.

7. What is the composition of your management team?
Select all that apply: Graduate / Head of unit / Head of service / Clinician nurse / Specialized clinician nurse / Trainer / Other.

8. Have you ever heard the word sepsis?
Single choice: Yes / No.

9. If yes: Have you ever had training on sepsis during or after your studies?
Single choice: Yes / No.

10. If yes: When was your last sepsis training?
Single choice: Less than six months ago / six months to one year ago / on year to two years ago / two years to three years ago / more than three years ago.

11. How do you estimate your knowledge of sepsis?
Single choice: Very good / Good / Average / Poor / Very poor.

12. How do you estimate your capacity to manage sepsis?
Single choice: Very good / Good / Average / Poor / Very poor.

**- Theoretical questions**

13. Sepsis is defined by …
Single choice: An infection and a systemic inflammatory response / an infection and hemodynamic instability / an infection and organ dysfunction / an infection and a bacteremia / an infection not responding to antibiotics.

14. Do you agree with this statement? Every infected patient should be monitored for sepsis.
Single choice: Strongly agree / Agree / Neutral / Disagree / Strongly disagree.

15. Do you agree with this statement? A new unexplained organ dysfunction should lead to investigation for infection.
Single choice: Strongly agree / Agree / Neutral / Disagree / Strongly disagree.

16. Do you agree with this statement? A patient treated by antibiotics cannot develop sepsis.
Single choice: Strongly agree / Agree / Neutral / Disagree / Strongly disagree.

17. Do you agree with this statement? Sepsis and septic shock are important causes of mortality and morbidity in Switzerland.
Single choice: Strongly agree / Agree / Neutral / Disagree / Strongly disagree.

18. Which of these factors increase(s) the risk of developing sepsis?
Select all that apply: Age / Hypothyroidism / Immunosuppression / Sepsis medical history / Active cancer.

19. Have you ever said to a physician “I think this patient has sepsis”?
Single choice. Yes / No.

20. Which clinical score is recommended as a predictor of mortality for infected patients?
Single choice. APACHE II score / SIRS score / qSOFA score / MEWS score / None of these scores / I do not know.

21. The quick Sequential Organ Failure Assessment (qSOFA) score is composed of.
Select all that apply. Temperature / Blood pressure / Heart rate / Leucocytes / Respiratory rate / Creatinine / Glasgow coma scale.

22. Which score defines sepsis clinically?
Single choice. SOFA score / APACHE II score / SIRS score / MEWS score / None of these scores / I do not know.

23. Sepsis mortality rate is.
Single choice, scale from 0 to 100%.

24. Septic shock mortality rate is.
Single choice, scale from 0 to 100%.

25. Do you agree with this statement? Sepsis is a medical emergency.
Single choice: Strongly agree / Agree / Neutral / Disagree / Strongly disagree.

26. According to the latest recommendations, when should the first diagnosis and therapeutical measures be taken when there is a suspicion of sepsis?
Single choice. In the first hour / In the first three hours / In the first six hours / In the first twelve hours / In the first twenty-four hours.

**- Clinical questions**

**Part one, qSOFA score**
27a. Medical specialties nurses. A 70-year-old patient admitted for an upper gastrointestinal bleed develops new-onset dyspnea and a productive cough. The physical exam is notable for right base crackles and vital signs are as follow: temperature 38.5°C, heart rate 97 beats per minute, respiratory rate 25/minute, blood pressure 111/78 mmHg, Glasgow Coma Scale 13/15. What is the patient’s qSOFA score?
Single choice. One / Two / Three / Four / I do not know.

27b. Surgical specialties nurses. A 65-year-old patient admitted for a hemicolectomy for a localized colon tumor develops fever and local pain at the operative site two weeks after the intervention. The physical exam shows localized abdominal tenderness and guarding and her vital signs are as follow: temperature 38.5°C, heart rate 97 beats per minute, respiratory rate 25/minute, blood pressure 111/78 mmHg, Glasgow Coma Scale 13/15. What is the patient’s qSOFA score?
Single choice. One / Two / Three / Four / I do not know.

27c. Gynecology/Obstetrics nurses. A 34-year -old patient develops fever 24 hours after a cesarean delivery. The physical exam shows tenderness at uterine mobilization and her vital signs are as follow: temperature 38.5°C, heart rate 97 beats per minute, respiratory rate 25/minute, blood pressure 111/78 mmHg, Glasgow Coma Scale 13/15. What is the patient’s qSOFA score?
Single choice. One / Two / Three / Four / I do not know.

27d. Emergency department nurses. A 70-year-old patient comes to ED because of a new-onset dyspnea and a productive cough. The physical exam shows right base crackles and vital signs are as follow: temperature 38.5°C, heart rate 97 beats per minute, respiratory rate 25/minute, blood pressure 111/78 mmHg, Glasgow Coma Scale 13/15.
What is the patient’s qSOFA score? Single choice. One / Two / Three / Four / I do not know.

**Part two, management**
28. The qSOFA score is composed of three clinical parameters: respiratory rate of 22/min or greater, altered mentation and systolic blood pressure of 100mmHg or less. Each of these parameters is worth one point and a qSOFA score of two or more predicts an adverse outcome in a hospitalised infected patient.
This patient has a respiratory rate higher than 22/min and altered mentation (GCS < 15) and his qSOFA score is 2.
What is (are) your next(s) step(s)?
Select all that apply. Immediately call the attributed physician / Draw blood and send it to the lab / Nothing / Draw blood cultures / Vital signs monitoring
