# Supplementary material for: Sepsis awareness and knowledge amongst nurses, physicians and paramedics of a tertiary care center in Switzerland: A survey-based cross-sectional study
Source: PLoS One. 2023 Jun 28;18(6):e0285151. doi: 10.1371/journal.pone.0285151 (PMC10306229; doi:10.1371/journal.pone.0285151)
Supplement: S2 File — (DOCX) [file pone.0285151.s005.docx]

**Survey translation, paramedics version

- Demographics**

1. What year were you born in?
Single choice with years from 1950 to 2004.

2. What is your gender?
Single choice: Male / Female/ Other.

3. How many years have you worked in healthcare?
Single choice: < 1 year / from 1 to 3 years / from 3 to 5 years / from 5 to 10 years / from 10 to 15 years / more than 15 years.

4. What is your profession?
Single choice: Nurse / Physician / Paramedic.

5. What is your education?
Single choice: Ambulance Technician / Paramedic / Nurse / Other.

6. Have you ever heard the word sepsis?
Single choice: Yes / No.

7. If yes: Have you ever had training on sepsis during or after your studies?
Single choice: Yes / No.

8. If yes: When was your last sepsis training?
Single choice: Less than six months ago / six months to one year ago / on year to two years ago / two years to three years ago / more than three years ago.

9. How do you estimate your knowledge of sepsis?
Single choice: Very good / Good / Average / Poor / Very poor.

10. How do you estimate your capacity to manage sepsis?
Single choice: Very good / Good / Average / Poor / Very poor.

**- Theoretical questions**

11. Sepsis is defined by …
Single choice: An infection and a systemic inflammatory response / an infection and hemodynamic instability / an infection and organ dysfunction / an infection and a bacteremia / an infection not responding to antibiotics.

12. Do you agree with this statement? Every infected patient should be monitored for sepsis.
Single choice: Strongly agree / Agree / Neutral / Disagree / Strongly disagree.

13. Do you agree with this statement? A new unexplained organ dysfunction should lead to investigation for infection.
Single choice: Strongly agree / Agree / Neutral / Disagree / Strongly disagree.

14. Do you agree with this statement? A patient treated by antibiotics cannot develop sepsis.
Single choice: Strongly agree / Agree / Neutral / Disagree / Strongly disagree.

15. Do you agree with this statement? Sepsis and septic shock are important causes of mortality and morbidity in Switzerland.
Single choice: Strongly agree / Agree / Neutral / Disagree / Strongly disagree.

16. Which of these factors increase(s) the risk of developing sepsis?
Select all that apply: Age / Hypothyroidism / Immunosuppression / Sepsis medical history / Active cancer.

17. Have you ever used to word “Sepsis” during a transmission?
Single choice: Yes / No.

18. Which clinical score is recommended as a predictor of mortality for infected patients?
Single choice. APACHE II score / SIRS score / qSOFA score / MEWS score / None of these scores / I do not know.

19. The quick Sequential Organ Failure Assessment (qSOFA) score is composed of.
Select all that apply. Temperature / Blood pressure / Heart rate / Leucocytes / Respiratory rate / Creatinine / Glasgow coma scale.

20. Sepsis mortality rate is.
Single choice, scale from 0 to 100%.

21. Septic shock mortality rate is.
Single choice, scale from 0 to 100%.

22. Do you agree with this statement? Sepsis is a medical emergency.
Single choice: Strongly agree / Agree / Neutral / Disagree / Strongly disagree.

23. According to the latest recommendations, when should the first diagnosis and therapeutical measures be taken when there is a suspicion of sepsis?
Single choice. In the first hour / In the first three hours / In the first six hours / In the first twelve hours / In the first twenty-four hours.

**- Clinical questions**

**Part one, qSOFA score**
24. You arrive to a patient’s home after a call from her husband. He tells you that she has been coughing for many days and developed fever this morning. Her vital signs are as follow: temperature 38.5°C, heart rate 97 beats per minute, respiratory rate 25/minute, blood pressure 111/78 mmHg, Glasgow Coma Scale 13/15.
What is the patient’s qSOFA score? Single choice. One / Two / Three / Four / I do not know.

**Part two, management**
25. The qSOFA score is composed of three clinical parameters: respiratory rate of 22/min or greater, altered mentation and systolic blood pressure of 100mmHg or less. Each of these parameters is worth one point and a qSOFA score of two or more predicts an adverse outcome in a hospitalized infected patient.
This patient has a respiratory rate higher than 22/min and altered mentation (GCS < 15) and his qSOFA score is 2.
What is (are) your next(s) step(s)?
Select all that apply: Rapidly transfer patient to ED / Reassure the husband and tell him to go to emergency department if his wife doesn’t get better / Reassure the husband and tell him to consult the family physician if his wife doesn’t get better / Start vital signs monitoring / No special measure.
