# Supplementary material for: Sepsis awareness and knowledge amongst nurses, physicians and paramedics of a tertiary care center in Switzerland: A survey-based cross-sectional study
Source: PLoS One. 2023 Jun 28;18(6):e0285151. doi: 10.1371/journal.pone.0285151 (PMC10306229; doi:10.1371/journal.pone.0285151)
Supplement: S5 File — (PDF) [file pone.0285151.s008.pdf]

Safe - PDF Ambulancier

Record ID ______________________

Quelle est votre année de naissance?

- ◯ 2004
- ◯ 2003
- ◯ 2002
- ◯ 2001
- ◯ 2000
- ◯ 1999
- ◯ 1998
- ◯ 1997
- ◯ 1996
- ◯ 1995
- ◯ 1994
- ◯ 1993
- ◯ 1992
- ◯ 1991
- ◯ 1990
- ◯ 1989
- ◯ 1988
- ◯ 1987
- ◯ 1986
- ◯ 1985
- ◯ 1984
- ◯ 1983
- ◯ 1982
- ◯ 1981
- ◯ 1980
- ◯ 1979
- ◯ 1978
- ◯ 1977
- ◯ 1976
- ◯ 1975
- ◯ 1974
- ◯ 1973
- ◯ 1972
- ◯ 1971
- ◯ 1970
- ◯ 1969
- ◯ 1968
- ◯ 1967
- ◯ 1966
- ◯ 1965
- ◯ 1964
- ◯ 1963
- ◯ 1962
- ◯ 1961
- ◯ 1960
- ◯ 1959
- ◯ 1958
- ◯ 1957
- ◯ 1956
- ◯ 1955
- ◯ 1954
- ◯ 1953
- ◯ 1952
- ◯ 1951
- ◯ 1950

Quel est votre genre?

- ◯ Masculin
- ◯ Féminin
- ◯ Intersexe

Depuis combien de temps travaillez-vous dans le domaine médical (sans compter les études)?	◯ Moins d'un an<br>◯ Entre 1 et 3 ans<br>◯ Entre 3 et 5 ans<br>◯ Entre 5 et 10 ans<br>◯ Entre 10 et 15 ans<br>◯ Plus de 15 ans
Quel est votre corps de métier?	◯ Infirmier.ère<br>◯ Ambulancier.ère<br>◯ Médecin
Quel est votre formation?	◯ Technicien.ne ambulancier.ère<br>◯ Ambulancier. ère<br>◯ Infirmier. ère<br>◯ Autre
Avez-vous déjà entendu le mot sepsis?	◯ Oui<br>◯ Non
Avez-vous déjà eu une formation sur le sepsis pendant ou après vos études?	◯ Oui<br>◯ Non
De quand date votre dernière formation sur le sepsis?	◯ Il y a moins de 6 mois<br>◯ Il y a moins d'1 an<br>◯ Il y a moins de 2 ans<br>◯ Il y a moins de 3 ans<br>◯ Il y a plus de 3 ans
Comment estimez-vous vos connaissances dans le domaine du sepsis?	◯ Très bonnes<br>◯ Bonnes<br>◯ Moyennes<br>◯ Mauvaises<br>◯ Très mauvaises
Comment estimez-vous votre capacité à gérer un sepsis?	◯ Très bonne<br>◯ Bonne<br>◯ Moyenne<br>◯ Mauvaise<br>◯ Très mauvaise
Le sepsis se définit par...	◯ Une infection et une réponse inflammatoire systémique<br>◯ Une infection et une instabilité hémodynamique<br>◯ Une infection et une dysfonction d'organe<br>◯ Une infection et une bactériémie<br>◯ Une infection et une non-réponse au traitement antibiotique

	Tout à fait d'accord	D'accord	Ni d'accord ni en désaccord	Peu d'accord	Pas du tout d'accord
A quel point êtes-vous d'accord avec cette affirmation? Tout patient infecté devrait être surveillé pour un éventuel sepsis	◯	◯	◯	◯	◯

A quel point êtes-vous d'accord avec cette affirmation? Toute nouvelle dysfonction d'organe inexpliquée devrait faire rechercher une infection ◯ ◯ ◯ ◯ ◯

A quel point êtes-vous d'accord avec cette affirmation? Un patient sous antibiothérapie ne peut pas développer un sepsis ◯ ◯ ◯ ◯ ◯

A quel point êtes-vous d'accord avec cette affirmation? Le sepsis et le choc septique sont des causes importantes de mortalité et de morbidité en Suisse ◯ ◯ ◯ ◯ ◯

Parmi ces facteurs, lesquels augmentent le risque de développer un sepsis?
(Plusieurs réponses possibles)

- ☐ L'âge
- ☐ L'hypothyroïdie
- ☐ L'immunosuppression
- ☐ Un antécédent de sepsis
- ☐ Un cancer actif

Avez-vous déjà utilisé le mot "sepsis" lors d'une transmission aux urgences?

- ◯ Oui
- ◯ Non

Dans quel contexte?

- ◯ Lors d'un transfert
- ◯ Lors d'une urgence
- ◯ Les deux

Quel score clinique est actuellement recommandé comme prédicteur de mortalité chez un patient infecté?

- ◯ Le score APACHE II
- ◯ Le score SIRS
- ◯ Le score qSOFA
- ◯ Le score MEWS
- ◯ Aucun de ces scores
- ◯ Je ne sais pas

Le score qSOFA (Quick Sepsis related Organ Failure Assessment) se compose de:
(Plusieurs réponses possibles)

- ☐ La température
- ☐ La tension artérielle
- ☐ La fréquence cardiaque
- ☐ Les leucocytes
- ☐ La fréquence respiratoire
- ☐ La créatinine
- ☐ Le score de Glasgow

Le taux de mortalité moyen du sepsis est de x %

(Place a mark on the scale above)

Le taux de mortalité moyen du choc septique est de x %

(Place a mark on the scale above)

	Tout à fait d'accord	D'accord	Ni d'accord ni et désaccord	Peu d'accord	Pas du tout d'accord
A quel point êtes-vous d'accord avec cette affirmation? Le sepsis est une urgence médicale	◯	◯	◯	◯	◯

Selon les recommandations les plus récentes, dans quel délai faut-il instaurer les premières mesures diagnostiques et thérapeutiques devant une suspicion de sepsis?	◯ 1h<br>◯ 3h<br>◯ 6h<br>◯ 12h<br>◯ 24h
Vous arrivez au domicile d'une patiente de 70 ans suite à l'appel de son mari. Il vous raconte qu'elle tousse depuis plusieurs jours et qu'elle a de la fièvre depuis ce matin. A l'examen clinique, ses constantes vitales sont les suivantes:<br>Température 38.5°C, fréquence cardiaque 97 battements par minute, fréquence respiratoire 25/min, tension artérielle 111/78 mmHg, Glasgow Coma Scale 13/15.<br>Quel est le score qSOFA de la patiente?	◯ 1<br>◯ 2<br>◯ 3<br>◯ 4<br>◯ Je ne sais pas
Le score qSOFA (quick Sepsis related Organ Failure Assessment) comprend trois paramètres cliniques valant chacun un point:<br>- Fréquence respiratoire > 22/min<br>- Tension artérielle systolique < 100 mmHg<br>- Glasgow Coma Scale < 15<br>Un qSOFA plus grand ou égal à 2 prédit un mauvais pronostique chez un patient infecté.<br><br>Ici, notre patiente a un qSOFA à 2 en raison de sa fréquence respiratoire supérieure à 22/min et de son Glasgow Coma Scale inférieur à 15.<br><br>Quelle(s) attitude(s) est/sont la/les plus appropriée(s)?<br>(Plusieurs réponses possibles)	☐ Transférer rapidement la patiente aux urgences<br>☐ Rassurer le mari de la patiente et lui dire de consulter immédiatement aux urgences si son état ne s'améliore pas<br>☐ Rassurer le mari de la patiente et lui dire de consulter son médecin traitant si son état ne s'améliore pas<br>☐ Instaurer une surveillance rapprochée des paramètres vitaux<br>☐ Pas de mesure particulière

Merci d'avoir répondu à ce questionnaire !

Le but du projet étant l'amélioration de la prise en charge du sepsis au sein de l'institution, nous vous recontacterons par email à la fin de la récolte des données afin de vous donner les réponses attendues. En nous basant sur les résultats du questionnaire, nous allons également travailler sur un module éducatif en ligne qui sera mis à votre disposition.
