# Supplementary material for: Sepsis awareness and knowledge amongst nurses, physicians and paramedics of a tertiary care center in Switzerland: A survey-based cross-sectional study
Source: PLoS One. 2023 Jun 28;18(6):e0285151. doi: 10.1371/journal.pone.0285151 (PMC10306229; doi:10.1371/journal.pone.0285151)
Supplement: S6 File — (PDF) [file pone.0285151.s009.pdf]

Safe - PDF Médecin

Record ID ______________________________

Quelle est votre année de naissance?

◯ 2004
◯ 2003
◯ 2002
◯ 2001
◯ 2000
◯ 1999
◯ 1998
◯ 1997
◯ 1996
◯ 1995
◯ 1994
◯ 1993
◯ 1992
◯ 1991
◯ 1990
◯ 1989
◯ 1988
◯ 1987
◯ 1986
◯ 1985
◯ 1984
◯ 1983
◯ 1982
◯ 1981
◯ 1980
◯ 1979
◯ 1978
◯ 1977
◯ 1976
◯ 1975
◯ 1974
◯ 1973
◯ 1972
◯ 1971
◯ 1970
◯ 1969
◯ 1968
◯ 1967
◯ 1966
◯ 1965
◯ 1964
◯ 1963
◯ 1962
◯ 1961
◯ 1960
◯ 1959
◯ 1958
◯ 1957
◯ 1956
◯ 1955
◯ 1954
◯ 1953
◯ 1952
◯ 1951
◯ 1950

Quel est votre genre?

◯ Masculin
◯ Féminin
◯ Intersexe

Depuis combien de temps travaillez-vous dans le domaine médical (sans compter les études)?	◯ Moins d'un an<br>◯ Entre 1 et 3 ans<br>◯ Entre 3 et 5 ans<br>◯ Entre 5 et 10 ans<br>◯ Entre 10 et 15 ans<br>◯ Plus de 15 ans
Quel est votre corps de métier?	◯ Infirmier.ère<br>◯ Ambulancier.ère<br>◯ Médecin
Quelle est votre fonction?	◯ Assistant.e<br>◯ Chef.fe de clinique<br>◯ Cadre

Dans quel service travaillez-vous?	◯ Service des urgences<br>◯ Service de médecine intensive adulte<br>◯ Service de médecine interne<br>◯ Service d'endocrinologie, diabétologie et métabolisme<br>◯ Service de gastroenérologie et d'hépatologie<br>◯ Service de gériatrie et réadaptation gériatrique<br>◯ Service d'immunologie et allergie<br>◯ Service de médecine génétique<br>◯ Service des maladies infectieuses<br>◯ Service de néphrologie<br>◯ Service de pneumologie<br>◯ Service de cardiologie<br>◯ Service d'angiologie<br>◯ Service d'hématologie<br>◯ Service de rhumatologie<br>◯ Service de neurologie<br>◯ Service de neuropsychologie et neuroréabilitation<br>◯ Centre Leenaards de la mémoire<br>◯ Service de soins palliatifs et de support<br>◯ Centre de transplantation d'organes<br>◯ Centre d'investigation et de recherche sur le sommeil<br>◯ Service de dermatologie et vénérologie<br>◯ Service de médecine préventive hospitalière<br>◯ Service d'anesthésiologie<br>◯ Service de chirurgie cardiaque<br>◯ Service de chirurgie thoracique<br>◯ Service de chirurgie viscérale<br>◯ Service de chirurgie vasculaire<br>◯ Centre de chirurgie septique<br>◯ Service d'orthopédie et de traumatologie<br>◯ Service de chirurgie plastique et de la main<br>◯ Division de médecine physique et de réhabilitation<br>◯ Service de neurochirurgie<br>◯ Service d'urologie<br>◯ Service d'oto-rhino-laryngologie<br>◯ Service de recherche chirurgicale<br>◯ Service de gynécologie et d'obstétrique<br>◯ Service de chirurgie pédiatrique<br>◯ Service d'oncologie médicale<br>◯ Service de radio-oncologie<br>◯ Service d'immuno-oncologie<br>◯ Service de radiodiagnostic et radiologie interventionnelle<br>◯ Département de psychiatrie<br>◯ Cellules plaies et cicatrisation
Avez-vous déjà entendu le mot sepsis?	◯ Oui<br>◯ Non
Avez-vous déjà eu une formation sur le sepsis pendant ou après vos études?	◯ Oui<br>◯ Non
De quand date votre dernière formation sur le sepsis?	◯ Il y a moins de 6 mois<br>◯ Il y a moins d'1 an<br>◯ Il y a moins de 2 ans<br>◯ Il y a moins de 3 ans<br>◯ Il y a plus de 3 ans

Comment estimez-vous vos connaissances dans le domaine du sepsis?	◯ Très bonnes<br>◯ Bonnes<br>◯ Moyennes<br>◯ Mauvaises<br>◯ Très mauvaises
Comment estimez-vous votre capacité à gérer un sepsis?	◯ Très bonne<br>◯ Bonne<br>◯ Moyenne<br>◯ Mauvaise<br>◯ Très mauvaise
Le sepsis se définit par...	◯ Une infection et une réponse inflammatoire systémique<br>◯ Une infection et une instabilité hémodynamique<br>◯ Une infection et une dysfonction d'organe<br>◯ Une infection et une bactériémie<br>◯ Une infection et une non-réponse au traitement antibiotique
Le choc septique se définit par un sepsis et...<br>(Plusieurs réponses possibles)	☐ Une instabilité hémodynamique nécessitant des vasopresseurs malgré un remplissage adéquat<br>☐ Un score SIRS de > 2 points<br>☐ Une bactériémie<br>☐ Une lactatémie à > 2 mmol/l<br>☐ Un score SOFA à > 10 points

	Tout à fait d'accord	D'accord	Ni d'accord ni en désaccord	Peu d'accord	Pas du tout d'accord
A quel point êtes-vous d'accord avec cette affirmation? Tout patient infecté devrait être surveillé pour un éventuel sepsis	◯	◯	◯	◯	◯
A quel point êtes-vous d'accord avec cette affirmation? Toute nouvelle dysfonction d'organe inexpliquée devrait faire rechercher une infection	◯	◯	◯	◯	◯
A quel point êtes-vous d'accord avec cette affirmation? Un patient sous antibiothérapie ne peut pas développer un sepsis	◯	◯	◯	◯	◯
A quel point êtes-vous d'accord avec cette affirmation? Le sepsis et le choc septique sont des causes importantes de mortalité et de morbidité en Suisse	◯	◯	◯	◯	◯

Parmi ces facteurs, lesquels augmentent le risque de développer un sepsis?<br>(Plusieurs réponses possibles)	☐ L'âge<br>☐ L'hypothyroïdie<br>☐ L'immunosuppression<br>☐ Un antécédent de sepsis<br>☐ Un cancer actif

Avez-vous déjà ajouté "sepsis" à votre liste de diagnostics?	◯ Oui<br>◯ Non
Quel score clinique est actuellement recommandé comme prédicteur de mortalité chez un patient infecté?	◯ Le score APACHE II<br>◯ Le score SIRS<br>◯ Le score qSOFA<br>◯ Le score MEWS<br>◯ Aucun de ces scores<br>◯ Je ne sais pas
Le score qSOFA (Quick Sepsis related Organ Failure Assessment) se compose de:<br>(Plusieurs réponses possibles)	☐ La température<br>☐ La tension artérielle<br>☐ La fréquence cardiaque<br>☐ Les leucocytes<br>☐ La fréquence respiratoire<br>☐ La créatinine<br>☐ Le score de Glasgow
Quel score permet de définir le sepsis en clinique?	◯ Le score SOFA<br>◯ Le score APACHE II<br>◯ Le score SIRS<br>◯ Le score MEWS<br>◯ Aucun de ces scores<br>◯ Je ne sais pas
Avez-vous déjà calculé un score SOFA pour un de vos patients?	◯ Oui<br>◯ Non
Le taux de mortalité moyen du sepsis est de x %	*(Place a mark on the scale above)*
Le taux de mortalité moyen du choc septique est de x %	*(Place a mark on the scale above)*

	Tout à fait d'accord	D'accord	Ni d'accord ni et désaccord	Peu d'accord	Pas du tout d'accord
A quel point êtes-vous d'accord avec cette affirmation? Le sepsis est une urgence médicale	◯	◯	◯	◯	◯

Selon les recommandations les plus récentes, dans quel délai faut-il instaurer les premières mesures diagnostiques et thérapeutiques devant une suspicion de sepsis?	◯ 1h<br>◯ 3h<br>◯ 6h<br>◯ 12h<br>◯ 24h

Une patiente de 70 ans hospitalisée pour hémorragie digestive haute développe une dyspnée ainsi qu'une toux productive à l'étage de médecine. A l'examen clinique, on retrouve des râles crépitants en base droite et ses constantes vitales sont les suivantes:
Température 38.5°C, fréquence cardiaque 97 battements par minute, fréquence respiratoire 25/min, tension artérielle 111/78 mmHg, Glasgow Coma Scale 13/15.
Quel est le score qSOFA de la patiente?

- ◯ 1
- ◯ 2
- ◯ 3
- ◯ 4
- ◯ Je ne sais pas

Une patiente de 70 ans est amenée aux urgences par son mari. Elle se plaint d'une dyspnée nouvelle ainsi que d'une toux productive. A l'examen clinique, on retrouve des râles crépitants en base droite et ses constantes vitales sont les suivantes:
Température 38.5°C, fréquence cardiaque 97 battements par minute, fréquence respiratoire 25/min, tension artérielle 111/78 mmHg, Glasgow Coma Scale 13/15.
Quel est le score qSOFA de la patiente?

- ◯ 1
- ◯ 2
- ◯ 3
- ◯ 4
- ◯ Je ne sais pas

Une patiente de 34 ans 2 geste 2 pare développe un état fébrile 24 heures après un accouchement par césarienne. A l'examen clinique, elle se plaint de douleurs à la mobilisation utérine et ses constantes vitales sont les suivantes:
Température 38.5°C, fréquence cardiaque
97 battements par minute, fréquence respiratoire 25/min, tension artérielle 112/78 mmHg, Glasgow Coma Scale 13/15.
Quel est le score qSOFA de la patiente?

- ◯ 1
- ◯ 2
- ◯ 3
- ◯ 4
- ◯ Je ne sais pas

Une patiente de 65 ans opérée d'une hémi colectomie pour un cancer du colon localisé développe de la fièvre ainsi qu'une douleur locale au niveau d'une des plaies opératoires 2 semaines après l'opération. A l'examen clinique, on retrouve une sécrétion nouvelle au niveau de la plaie. Les constantes vitales sont les suivantes:
Température 38.5°C, fréquence cardiaque
97 battements par minute, fréquence respiratoire 25/min, tension artérielle 112/78 mmHg, Glasgow Coma Scale 13/15.
Quel est le score qSOFA de la patiente?

- ◯ 1
- ◯ 2
- ◯ 3
- ◯ 4
- ◯ Je ne sais pas

Le score qSOFA (quick Sepsis related Organ Failure Assessment) comprend trois paramètres cliniques valant chacun un point:<br>- Fréquence respiratoire > 22/min<br>- Tension artérielle systolique < 100 mmHg<br>- Glasgow Coma Scale < 15<br>Un qSOFA plus grand ou égal à 2 prédit un mauvais pronostique chez un patient infecté.<br><br>Ici, notre patiente a un qSOFA à 2 en raison de sa fréquence respiratoire supérieure à 22/min et de son Glasgow Coma Scale inférieur à 15.<br><br>Quelles investigations complémentaires sont appropriées?<br>(Plusieurs réponses possibles)	☐ Des hémocultures<br>☐ Une surveillance rapprochée des constantes vitales<br>☐ Une prise de sang et une gazométrie<br>☐ Un électroencéphalogramme<br>☐ Une imagerie afin de localiser un éventuel foyer infectieux
Un laboratoire montre une thrombopénie à 88,000 thrombocytes par microlitre. Le score de Glasgow étant toujours à 13/15, le score SOFA de la patiente s'élève à 3 points.<br>Quelle prise en charge est la plus appropriée?<br>(Plusieurs réponses possibles)	☐ S'assurer de la présence de deux voies veineuses périphériques<br>☐ Administrer des corticoïdes<br>☐ Administrer des antibiotiques à large spectre dirigés sur le diagnostic de suspicion<br>☐ Administrer des immunoglobulines intraveineuses (IGIV)<br>☐ Effectuer une réanimation liquidienne et optimiser la volémie

Merci d'avoir répondu à ce questionnaire !

Le but du projet étant l'amélioration de la prise en charge du sepsis au sein de l'institution, nous vous recontacterons par email à la fin de la récolte des données afin de vous donner les réponses attendues. En nous basant sur les résultats du questionnaire, nous allons également travailler sur un module éducatif en ligne qui sera mis à votre disposition.
