# Supplementary material for: Sepsis awareness and knowledge amongst nurses, physicians and paramedics of a tertiary care center in Switzerland: A survey-based cross-sectional study
Source: PLoS One. 2023 Jun 28;18(6):e0285151. doi: 10.1371/journal.pone.0285151 (PMC10306229; doi:10.1371/journal.pone.0285151)
Supplement: Attachment — Submitted filename: 20221025_Reviewers_comments.pdf [file pone.0285151.s012.pdf]

Service des maladies infectieuses
Bâtiment hospitalier
Rue du Bugnon 46
1011 Lausanne

Editor comments

None.

Reviewer 1

Reviewer #1: I thank the authors for their clear and detailed responses and for considering all suggestions.

We thank the reviewer for his evaluation.

Reviewer 2

Reviewer #2: Thank you for your great effort. The authors have satisfactorily addressed all of my comments.

Hope I can see your publication soon!

We are very thankful for the reviewer's kind words.

Reviewer #3:

Reviewer #3: Thank you for paying close attention to the recommended changes. In my estimation, most of them have been well addressed. A few places remain unclear and need revision:

- *On page 4, line 89, gaps in knowledge among medical and paramedical staff were mentioned and referenced. Nurses should also be added to the list as several of your studies include nurses. An additional study, should you want it, that identifies similar knowledge gaps to yours, is Storozuk et al. (2019). A survey of sepsis knowledge among Canadian emergency department registered nurses. Australasian Emergency Care, 22, 119-125. https://doi.org/10.1016/j.auec.2019.01.007*

We thank the reviewer for this comment. We had unfortunately used the Britannica definition of paramedical staff which includes nurses. We have now added specifically the Word "nursing staff" and added the additional reference.

canton de vaud

Service des maladies infectieuses
Bâtiment hospitalier
Rue du Bugnon 46
1011 Lausanne

• On page 5 (line 106), the phrase "active sepsis programme" was used. It is not clear whether this is an education programme or a clinical practice program. Please clarify.

We thank the reviewer for this comment. We have clarified the text:

At the time of the survey, no department had an active education or clinical practice sepsis programme.

• Method – the word "screened" and the phrase "screened out" do not make sense to me. They do not translate well for an international audience and I do not know what is meant. Please use a different word/phrase.

We would ask the reviewer to clarify which part of the text is referred to as we did not find the part of the manuscript in question.

• Writing – There are still quite a few small but important places throughout the manuscript where copy editing for grammar revisions and English language are needed, primarily for apostrophes, correct verb tense, and preposition use.
Again, overall, this is a timely and interesting QI study, that adds to the international literature.

We thank the reviewer fort her comment as well. The text has been reviewed by a English linguistic expert at UNIL (Nicolas Meylan) and multiple changes have been introduced through out to improve the language. The text has, we hope, gained in clarity and legibility.
