# Supplementary material for: Sepsis awareness and knowledge amongst nurses, physicians and paramedics of a tertiary care center in Switzerland: A survey-based cross-sectional study
Source: PLoS One. 2023 Jun 28;18(6):e0285151. doi: 10.1371/journal.pone.0285151 (PMC10306229; doi:10.1371/journal.pone.0285151)
Supplement: Attachment — Submitted filename: Review comments.docx [file pone.0285151.s010.docx]

Sepsis awareness of healthcare professionals is an important factor affecting patient survival rates. Therefore, the results obtained from the study will guide the preparation of educational content for the diagnosis and management of sepsis.

There is a need for explanatory information about the sampling method and the questionnaire form in the article.

Suggestions to increase the intelligibility of the article are given below.

**Background**

Page 3, line 63. The recommendations of the “2021 Sepsis Survival Campaign Guidelines” should be added and these guidelines should be reflected throughout the article.

**Methods**

Page 4, line 81-83. Was sample calculation used in the research? Used my snowball method? Was consent obtained from the participants in the online online survey? How was the privacy of the participants protected? Information must be added.

Are sepsis care protocols used in any unit of the hospital when specifying the universe characteristics? It must be disclosed.

*Measures*

Page 4, line 88-89. Were only bibliographies 22 and 23 used when preparing the measurement tool? In this sense, it is understood that quite old and limited resources are used.

Page 4, line 90-96. Which method was used to validate the questionnaire used in the study? Has scope validity index calculation been used for this? What was the expertise of the focus groups on the subject? It should be explained.

Page 4, line 95,96. Nurses are at a key point in delirium diagnosis as well as management. Why weren't nurses' management knowledge and skills measured? It should be explained.

*Data collection and recruitement*

Page 5. How were the participants prevented from repeating the online survey? It should be explained.

**Participants**

Page 6, line 138-141. Why was there no stratification in terms of representing the universe among occupational groups? The participation rate of paramedics is very high compared to other groups. The results of the study will be affected by this situation. The number of doctors is misspelled in Figure B (438!).

*Definition, detection and diagnosis of sepsis*

Page 8. If the sepsis education level of the participants and the experience of encountering a sepsis case were also measured, the power of the study would increase even more. (Can be specified in the limitations of the study)

Page 8, line 178. SOFA > 10 points. What source is this score based on?

*Factors associated with sepsis awareness*

Page 9, line 201- 208. A table can be given to increase the comprehensibility of the results obtained from "Univariate logistic regression models".

**Discussion**

The use of q-SOFA was recommended for diagnosing sepsis outside of intensive care units. The final guide discusses its reliability. For this reason, the use of q-SOFA in diagnosing sepsis should be discussed specifically for occupational groups working in intensive care and other fields.

**References**

The number of current resources can be increased.

**Figure 5**

Is the case information given here a case for sepsis? Blood pressure does not suggest sepsis! If different information about the case is not given, sepsis may not come to mind first.
