# Supplementary material for: Sepsis awareness and knowledge amongst nurses, physicians and paramedics of a tertiary care center in Switzerland: A survey-based cross-sectional study
Source: PLoS One. 2023 Jun 28;18(6):e0285151. doi: 10.1371/journal.pone.0285151 (PMC10306229; doi:10.1371/journal.pone.0285151)
Supplement: Attachment — Submitted filename: 20221220_SAfE_Manuscript_PLoS_Point-by-Point Response.docx [file pone.0285151.s013.docx]

*Reviewer #4:*

*The authors have not completely addressed all comments. The authors ignore our previous request of "A response to reviewers point by point is needed. Please copy and paste the changes in the manuscript to the response file too and indicate the page and line number".*

*1-Target participants should be presented in the title. What are local paramedics?*

The title of the manuscript was modified as requested by the reviewer and now reads **“Sepsis awareness and knowledge amongst nurses, physicians and paramedics of a tertiary care center in Switzerland: a survey-based cross-sectional study”**. Local paramedics are emergency medical services personnel providing emergency pre-hospital medical care on patient’s site and during transportation to hospital by ambulance. This has been clarified on line 35 page 94, line 94 and 97 page 5, by replacing the text “..local paramedics..” with “..paramedics transporting patients to our hospital”.

*2-Awareness is completely different from knowledge. There are several questions for knowledge and awareness, how did the author evaluate the level of knowledge and awareness? The authors need to define the outcomes of the study too.*

We thank the reviewer for this comment. We evaluated awareness based on the perceptions of the participant familiarity with the word sepsis. We assessed knowledge of sepsis based on questions focusing on the participants’ information/proficiency/skills on the definition, epidemiology, scores and management of sepsis. The words “awareness” and “knowledge” are integrated into the title of the article. This was also specified in the abstract “*Measured outcomes included professionals’ demographics (age, profession, seniority, unit of activity) and quantification of prior sepsis education, awareness and knowledge of sepsis assessed based on the information/proficiency/skills on the epidemiology, scores, definition and management of sepsis*”. Likewise, we modified the text in the methods (line 115-119 page 6) that reads “*The final survey contained questions on participants’ demographic characteristics (5/7/6 questions for nurses/paramedics/physicians), awareness was characterized by questions on sepsis perception and data on continuing education (3/3/3 questions) and self-evaluation of sepsis knowledge and clinical management (2/2/2 questions); the participants’ knowledge was characterized by questions on the definition, assessment scores, the epidemiology (11/12/14 questions) and the management of sepsis (4/4/5 questions)”*. Accordingly, the results section is now partitioned into participants, awareness and knowledge.

*3-Please cite the source of reporting checklist. The authors do not actually describe all information listed in the checklist such as testing of questionnaire, translation methods of questionnaire, providing questionnaire...*

In revision, we provide the reference of the CROSS checklist used for survey reporting (Sharma A et al. A Consensus-Based Checklist for Reporting of Survey Studies (CROSS). J Gen Intern Med. 2021 Oct;36(10):3179-3187. doi: 10.1007/s11606-021-06737-1.) The questionnaire was built *de novo* by the authors from references 9, 14, 24, and 25 (lines 103-104 page 5 of the manuscript). It was tested and revised through focus groups conducted with each professional category involved (lines 106-110 page 5 of the manuscript). The questionnaire and the answers to the questionnaire were written in French. In line 121-122 of the manuscript, we indicated that the questionnaire was translated into English by one of the authors and revised by another for publication. The survey was translated internally (JR, SM) but was reviewed externally by colleagues from the Brighton and Sussex University Hospital’s Intensive Care Unit. The English version of the survey is in the supplementary material. We have now added the survey in French.

*4-Please describe selection criteria. Did the authors exclude trainees?*

We have clarified selection criteria as specified on line 124-133, page 6 ” …including registered nurses, and physicians, including medical residents and fellows having graduated from medical school and who were in training for board certification in a medical specialty and attendings, issued from all departments (Emergency department (ED), intensive care unit (ICU), Medicine, Paramedic, Psychiatry, and Surgery) and professions (paramedics, nurses and physicians) in order to achieve maximum representativity of LUH staff considered as HCPs. Pediatrics and neonatology staff (not covered by Sepsis-3 consensus definitions) as well as nurses and physicians not in daily contact with patients (i.e., those working in research teams or in administration) were excluded. For paramedics, we included those transporting patients to LUH. **Undergraduate trainees were excluded**.”

*5-Please explain how to calculate the response rate at 91.8%? does it represent the whole population? why did the authors only contact 1216 participants among 4417 potential health care professionals?*

We favored an in person survey sampling to gain higher quality in participant response (e.g. taking the survey online with basic questions had a risk of participants looking up the definition online). To do so, we accessed HCPs mostly during continuing education rounds on their worksite. This meant that we actually got to meet with 1216 HCP out of the total 4417 HCP of LUH. Please bear in mind that the study, while designed in 2019, was performed during the COVID-19 pandemic when continuing education was severely impacted by social distancing measures. Due to strenuous work conditions, we only had specific time points for interviewing healthcare professionals. The participation rate thus represents the fraction of HCPs who were asked to participate. This is explained on lines 133-143, page 7 and in figure 1.

*6-Univariable regression analysis (NOT univariate) is not good enough to avoid confounders in association analysis. A multivariable regression analysis should be performed. These analyses should be presented in a table.*

We thank the reviewer for this important point. In our revision, we have replaced the univariable analysis with a multivariable analysis now provided in the abstract (page 3 and lines 51-55) and results section (i.e. on page 12 and lines 240-250 and in Table 3). We proceeded with multivariable regression analyses for each healthcare worker stratum.

*7-Values of odds ratio should have one more digit in the values.*

Odds ratio values were changed throughout the manuscript as requested by the reviewer.
